# Assessment of Electromagnetic Exposure Levels for Humans from Electric Vehicle DC Charging Stations

**DOI:** 10.3390/s25185735

**Published:** 2025-09-14

**Authors:** Shaowen Dong, Mai Lu

**Affiliations:** Key Laboratory of Opto-Electronic Technology and Intelligent Control of Ministry of Education, Lanzhou Jiaotong University, 88 West Anning Road, Anning District, Lanzhou 730070, China

**Keywords:** DC charging station, electric vehicle, realistic human model, equivalent radiation source, transformer, electromagnetic exposure, COMSOL multiphysics

## Abstract

The potential health risks of DC charging piles to human health were investigated by quantifying the internal electromagnetic exposure level. In this study, the transformer in the DC/DC circuit of a DC charging pile was selected as the radiation source, and two realistic human models (adult and child) were used as exposure subjects. A simulation model, including the vehicle body, charging pile, and transformer, was established using COMSOL(COMSOL Multiphysics 6.2) Multiphysics software to calculate the magnetic induction intensity (**B**-field) and electric field intensity (**E**-field) in various organs at distances of 0.1 m, 0.3 m, and 0.6 m from the charging pile. The results show that at 0.1 m, the peak **B**-field (1.91 µT) and **E**-field (447 mV/m) in the adult body were 1.91% and 2.07% of the ICNIRP occupational exposure limits, respectively, and 7.07% and 4.14% of the public exposure limits. For the child model, the peak electromagnetic exposure levels (2.31 µT and 259 mV/m) were only 8.56% and 2.40% of the public limits. Further evaluation of exposure levels for in-vehicle occupants during charging showed that the peak **B**-field and **E**-field for an adult driver and a child in the front passenger seat were 0.0225 × 10^−2^ µT, 0.0237 × 10^−2^ µT, 5.81 mV/m, and 5.82 mV/m, respectively, far below the ICNIRP public limits. Additionally, analyses at multiple frequency bands (85 kHz, 90 kHz, and 95 kHz) under a typical scenario (adult at 0.1 m from the charging pile) revealed that the **B**-field in the human body decreased with increasing frequency, while the **E**-field showed minimal variation due to shielding effects. All electromagnetic exposure levels were below both ICNIRP public and occupational limits, indicating the broad applicability of the results. Under normal operating conditions of DC charging piles, the electromagnetic exposure from the DC/DC transformer fully complies with safety standards and poses no threat to human health. This study provides a scientific basis for alleviating public concerns about the health risks of electromagnetic radiation from DC charging piles for electric vehicles.

## 1. Introduction

With the gradual depletion of traditional energy sources and the increasing severity of environmental pollution [1,2], various sectors are actively seeking solutions for energy conservation and storage [3]. Electric vehicles (EVs), due to their high efficiency and zero-emission characteristics, have gained widespread adoption globally [4,5]. To meet the growing demand for EV charging, countries worldwide are accelerating the development of charging infrastructure [6,7,8]. Direct current (DC) charging stations convert alternating current (AC) to DC to directly charge EV batteries, offering higher charging efficiency and faster speeds [9]. Owing to their rapid charging advantages, DC charging stations are widely deployed in public charging areas, such as highway service zones and urban fast-charging stations [10,11,12]. The establishment of charging stations provides convenient charging options for EV users and supports long-distance travel, further promoting the development of the new energy vehicle industry.

DC charging stations employ a modular design, where multiple charging units are connected in parallel to expand charging capacity. Modular units, composed of components such as rectifiers, transformers, and diodes, can be flexibly configured to meet diverse voltage requirements and enhance charging speed [9,13]. Among the components responsible for voltage conversion and electrical isolation in DC charging stations, the transformer is one of the components operating at the highest switching frequency. Due to sudden voltage changes, transformers generate high-frequency pulse signals within a short time [14], serving as a primary source of magnetic field radiation in the charging module. These pulse signals radiate electromagnetic fields into the surrounding environment, posing a significant source of electromagnetic interference (EMI) and potentially introducing various health risks to humans [15,16]. Therefore, further investigation into the potential impacts of electromagnetic environments on human health is of critical importance and will contribute to the development of relevant standards.

With the widespread adoption of electric vehicles (EVs), concerns regarding the safety of their electromagnetic environment have garnered increasing attention. Vassilev et al. [17] investigated magnetic fields inside eight different EVs and performed exposure calculations using the ICNIRP “weighted peak” method. Yang et al. [18] monitored the electromagnetic environment inside EV cabins over an extended period, concluding that magnetic fields within EVs remain largely unchanged despite prolonged driving or regular maintenance. Bazhynov et al. [19] found that the magnetic field induction intensity inside EV cabins during charging is significantly lower than that during driving. Concha et al. [20] used finite element simulations, measurements, and simplified analytical approximations to determine that magnetic fields generated by battery packs are below the ICNIRP reference levels. Hasselgren et al. [21] investigated the sensitivity of components and power line communication signals under varying noise intensities and frequency combinations, proposing solutions to enhance system immunity. Lennerz et al. [22] measured magnetic fields from EV charging stations and associated cables, finding that high-power chargers do not pose risks to patients with implanted cardiac pacemakers. Research on electromagnetic exposure during EV charging has primarily focused on wireless charging systems. Park et al. [23] examined human exposure levels in various scenarios involving an 85 kHz wireless power transfer (WPT) system for EVs, including cases with and without shielding and with aligned or misaligned transmitters and receivers. Zhao et al. [24] developed models of wireless charging systems and human bodies at different operating frequencies and power outputs, investigating the safe distance between humans and the charging system during wireless EV charging. Laakso et al. [25] studied electromagnetic exposure from leakage magnetic fields of wireless power transfer systems during EV wireless charging. Regarding safe distances between humans and radiation sources, Lu et al. [26] used the impedance method to calculate magnetic induction intensity and induced electric fields in the human body at 144 relative positions with respect to an H-coil for transcranial magnetic stimulation (TMS) caregivers, concluding that a minimum distance of 100 cm from the H-coil is required to ensure safety.

With the widespread deployment of charging stations, the electromagnetic environment and electromagnetic exposure issues associated with charging stations have attracted significant attention from researchers. He et al. [27] identified that electromagnetic interference (EMI) signals produced by DC/DC power converters are the primary sources of EMI in onboard charging systems. Paolo et al. [28] measured harmonics generated during the connection between EVs and charging stations, revealing that Level 3 charging stations produce more harmonic noise compared to Level 2 stations. Mazurek et al. [29] elucidated the characteristics of conducted disturbances and current harmonic levels generated by charging stations, as well as their impact on power quality. Nacu et al. [30] reduced harmonics to acceptable levels under IEC 61,000 standards by incorporating an LCL filter at the inverter output leads. Zheng et al. [31] summarized the working principles of DC charging stations and investigated the sources of EMI and methods for its suppression. Germana Trentadue et al. [32] measured low-frequency magnetic fields emitted by five fast-charging devices under charging and standby conditions using a magnetic field probe analyzer. Trentadue et al. [33] measured magnetic induction intensity from two high-power split-type DC charging systems, finding that measurements at certain points exceeded limits, but the electromagnetic environment measured at an increased distance from the charging facility complied with standards. Hongguk Bae et al. [34] assessed electromagnetic field exposure from six EV chargers, observing that the magnetic field of fast chargers in operation increases with charging current.

During the operation of DC charging stations, the rapid voltage transformations in the DC/DC circuit transformer within the charging module generate electromagnetic fields that may pose safety risks to personnel. This study quantitatively evaluates electromagnetic exposure for occupational workers and the general public of different age groups at distances of 0.1 m, 0.3 m, and 0.6 m from DC charging stations. It examines the electromagnetic exposure levels for an adult in the driver’s seat and a child in the front passenger seat during EV charging. Additionally, the study assesses electromagnetic exposure levels across different frequency bands in a typical scenario (an adult standing 0.1 m from the charging station). The findings of this study demonstrate that electromagnetic radiation from DC charging stations does not pose a health risk to humans, providing evidence to alleviate public concerns regarding electromagnetic exposure from EV DC charging stations.

## 2. Model Construction

Electromagnetic dosimetry [35] is a discipline that studies the effects of electromagnetic fields on biological systems, enabling precise quantification of electromagnetic exposure levels within organisms and evaluation of the safety of electromagnetic environments [36]. When investigating the distribution of electromagnetic fields in biological tissues, electromagnetic dosimetry methods can address the limitations of epidemiological surveys [37], direct measurement techniques [38], and Monte Carlo methods [39], which often fail to accurately assess human electromagnetic exposure levels. Based on electromagnetic dosimetry, this study investigates the operational characteristics of transformers in the DC/DC circuits of electric vehicle (EV) DC charging stations. Using finite element software, the magnetic induction intensity (**B**-field) and induced electric field intensity (**E**-field) were calculated for major organs of adults and children at various distances from the charging station and in the driver’s seat (adult) and front passenger seat (child) during EV charging in the 80 kHz frequency band. Additionally, electromagnetic exposure levels in the human body were estimated for a typical exposure scenario (a person standing 0.1 m from the charging station) across different frequencies. The numerical results were compared with the electromagnetic exposure limits outlined in the ICNIRP guidelines to assess the electromagnetic exposure levels for different individuals in this electromagnetic environment.

### 2.1. Human Model Description

**Figure 1:**
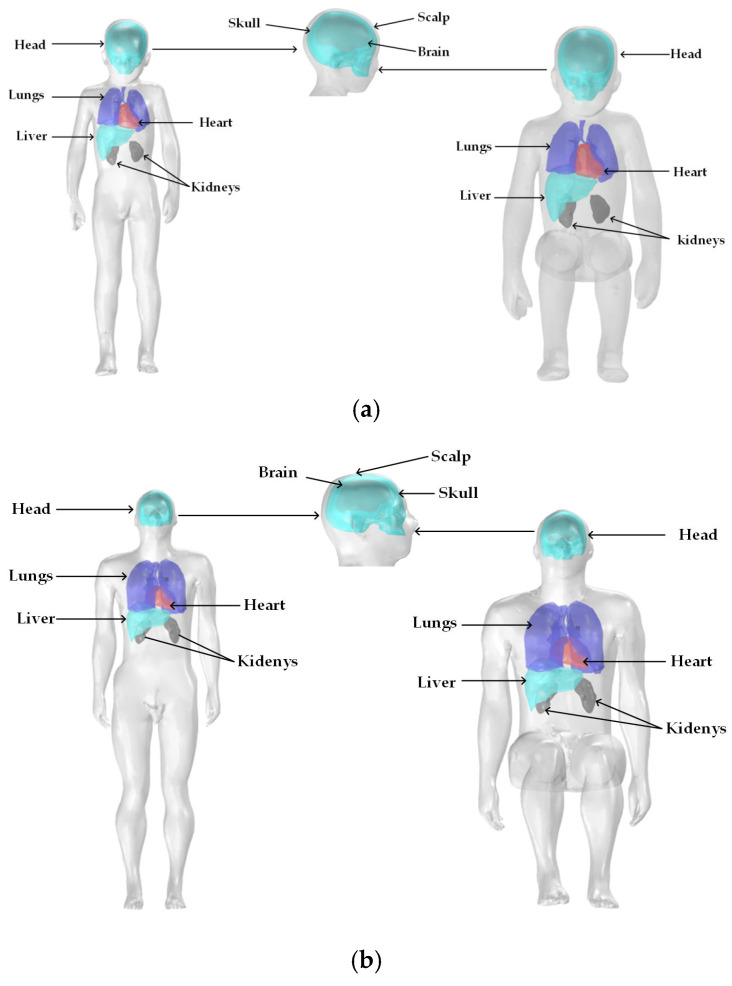
Human body models in standing and seated postures: (**a**) Adult, (**b**) Child.

The models used in this study include an adult male, Duke, and a boy, Thelonious. Duke has a standing height of 1.77 m, while Thelonious has a standing height of 1.15 m [40]. Based on the original anatomical models, several major organs and tissues were extracted, including the scalp, skull, brain, heart, liver, kidneys, and lungs, with the brain modeled as a combination of white and gray matter. The standing models were modified into seated postures using three-dimensional modeling software, as shown in the accompanying Figure 1.

### 2.2. DC Charging Station Model

**Figure 2:**
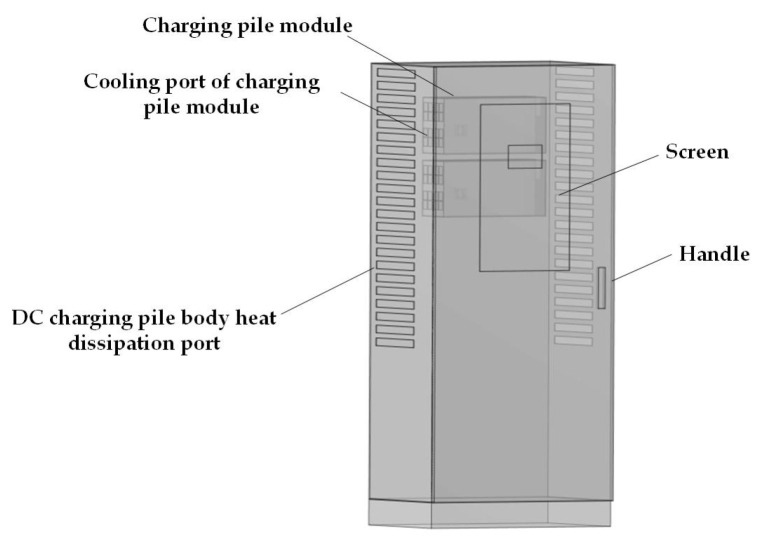
Model of the DC charging station.

**Figure 3:**
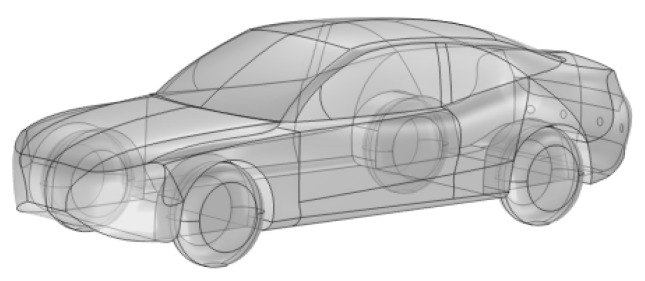
Model of the vehicle.

In this study, a DC charging station model was developed based on the actual dimensions of a commercially available 120 kW integrated DC charging station. The dimensions of the DC charging station are 800 mm × 500 mm × 1700 mm, as shown in Figure 2. The outer casing of the DC charging station is made of a 3 mm thick steel plate, with a 4 mm thick screen embedded in the center of the cabinet door. Inside the DC charging station, four 30 kW charging modules are installed, which are the core components and the primary sources of electromagnetic radiation. Each module has dimensions of 457 mm × 84 mm × 218 mm, with a steel casing of 2 mm thickness. The horizontal distance between two parallel charging modules is 2 mm, and the vertical distance between two vertically aligned modules is 20 mm. Both the DC charging station and its modules are equipped with ventilation openings to meet the heat dissipation requirements. Additionally, a pure electric vehicle (EV) model was established based on the actual dimensions of an EV. As shown in Figure 2 and Figure 3, with a body size of 5495 mm × 2200 mm × 1672 mm and a body material of aluminum alloy. The model also includes a transformer, human models, and a concrete platform.

**Figure 4:**
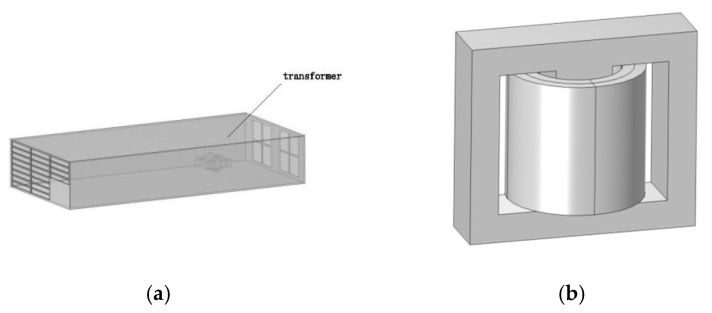
DC charging station module and transformer. (**a**) Relative position of the transformer within the charging station module; (**b**) equivalent model of the transformer.

The circuit within the charging module is complex, with the transformer being one of the components operating at the highest switching frequency, primarily serving the functions of electrical isolation and voltage regulation. In this study, the transformer is considered the primary radiation source in the DC charging station. To simplify computations while preserving key electromagnetic characteristics, as shown in Figure 4, the transformer was modeled as a magnetic core with two layers of windings, with dimensions of 48 mm × 28.8 mm × 42 mm. Based on the technical specifications of a 30 kW charging module from a specific brand obtained through market research, the turn ratio of the primary to secondary windings is 11:5, and the transformer’s switching frequency ranges from 80 kHz to 250 kHz, with an input voltage of 650 V. This study evaluates electromagnetic exposure for different individuals at transformer switching frequencies of 80 kHz, 85 kHz, 90 kHz, and 95 kHz.

**Figure 5:**
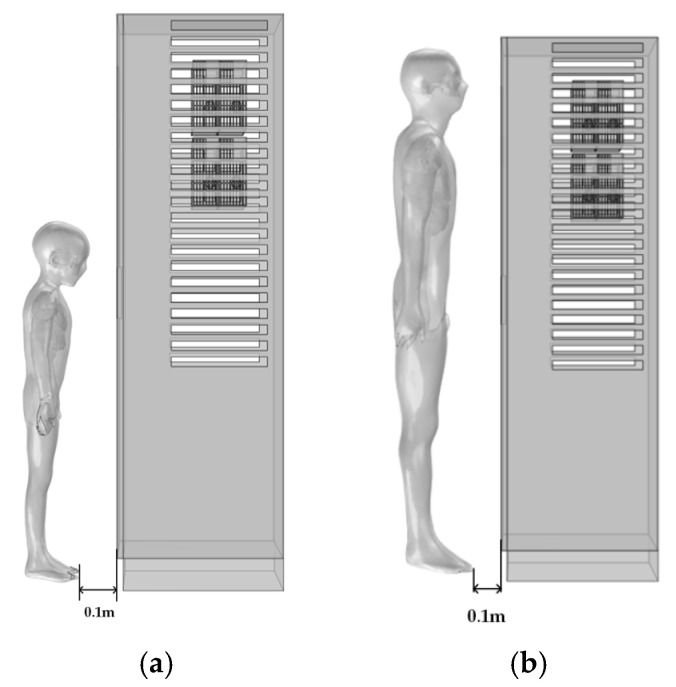
Human models located 0.1 m in front of the DC charging station. (**a**) Child model; (**b**) Adult model.

**Figure 6:**
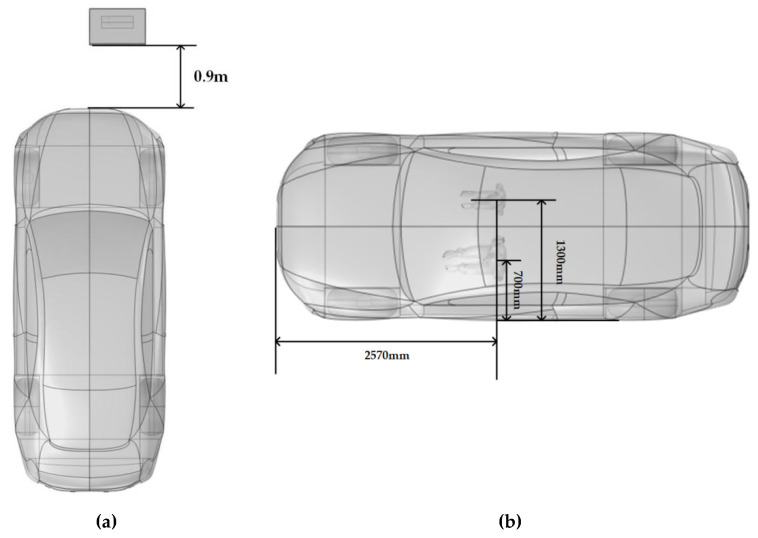
Relative spatial arrangements in the simulation: (**a**) Configuration of the EV and DC charging station; (**b**) Positions of the adult and child models within the vehicle.

Electromagnetic exposure levels were calculated for an adult and a child positioned directly in front of the DC charging station at distances of 0.1 m, 0.3 m, and 0.6 m, Figure 5 shows a scene with a child and an adult standing 0.1 meters from a charging station. Additionally, based on the configuration of an actual DC charging station, electromagnetic exposure values were determined for an adult driver in the driver’s seat and a child in the front passenger seat. As shown in Figure 6, with the vehicle located approximately 0.9 m vertically from the DC charging station. The vertical distance from the vehicle’s front to the driver (adult) and front passenger (child) is approximately 2570 mm, with the vertical distance from the left vehicle door to the driver’s body being approximately 700 mm and to the front passenger’s body approximately 1300 mm.

The dielectric constants and conductivities of materials, including aluminum alloy, tempered glass, copper, and ferrite, as reported in references [41,42], were applied. The conductivity and dielectric parameters of the primary materials used in the DC charging station and electric vehicle are presented in the Table 1.

### 2.3. Dielectric Parameters of Human Tissue

When conducting electromagnetic exposure assessments, appropriate dielectric parameters must be assigned to each organ and tissue. The dielectric properties of tissues exhibit frequency-dependent dispersion, resulting in variations in dielectric parameters across different frequencies [43]. In 1996, Gabriel et al. proposed a fourth-order Cole-Cole mathematical model [44,45,46], which can be used to calculate the conductivity and permittivity of biological tissues over a frequency range from 1 Hz to 100 GHz.

(1) $${\hat{\varepsilon }}_{r}=\varepsilon_{r}^{\prime }-j\varepsilon_{r}^{\prime \prime }=\varepsilon_{r\infty }+\sum_{n=0}^{4}\frac{\Delta \varepsilon_{n}}{1+{\left(j\omega \tau_{n}\right)}^{1-\alpha }}+\frac{\sigma_{i}}{j\omega \varepsilon_{0}}$$

In this model, ${\hat{\varepsilon }}_{r}$ represents the complex relative permittivity. The real part of ${\hat{\varepsilon }}_{r}$, denoted as $\varepsilon_{r}^{\prime }$, is commonly referred to as the relative permittivity. The imaginary part, $\varepsilon_{r}^{\prime \prime }$ is also known as the loss factor. The parameter $\Delta \varepsilon_{n}$ represents the change in relative permittivity, $\tau_{n}$ denotes the central relaxation time, and α is the distribution parameter, typically ranging between 0 and 1. $\sigma_{i}$ is the ionic conductivity (S/m), $\varepsilon_{r\infty }$ is the relative permittivity at optical frequencies, ω is the angular frequency (rad/s), which can be derived from the linear frequency, and $\varepsilon_{0}$ = 8.854187817 × 10^−12^ F/m is the permittivity of free space.

Using the fourth-order Cole-Cole model [46], the dielectric constant and conductivity of various human tissues at different frequencies were calculated. This study focuses on the distribution of electric and magnetic fields across various human organs. Table 2 presents the dielectric properties and conductivities of different organs at a frequency of 80 kHz. For the brain, the values of conductivity and permittivity are averaged from those of white matter and gray matter.

### 2.4. Numerical Methods

To address the complexities of the electromagnetic environment, the electromagnetic field generated by a high-frequency transformer under high-voltage input was simulated using COMSOL Multiphysics 6.2. In the Magnetic Fields interface, the governing equations were defined to solve Maxwell’s equations under the specified conditions. Maxwell’s equations are given as follows:

(2) $$\nabla \times H=J+\frac{\partial D}{\partial t}=J$$

(3) $$\nabla \times E=-\frac{\partial B}{\partial t}$$

(4) ∇·D=ρ

(5) ∇·B=0

where H is the magnetic field intensity (A/m), J is the induced current density (A/m^2^), E is the electric field intensity (V/m). D is the electric flux density (C/m^2^), and B is the magnetic induction intensity (T). ρ represents the volume charge density (V/m^3^). Assuming that the electromagnetic properties of biological tissues are linear and that a medium is present, the following three constitutive relations describe the medium within Maxwell’s equations:

(6) B=μH

(7) $$D=\varepsilon_{0}\varepsilon_{r}E$$

(8) J=σE

where μ is the magnetic permeability (H/m); $\varepsilon_{0}$ is the permittivity of free space (F/m), with a value of 8.85 × 10^−12^; $\varepsilon_{r}$ is the relative permittivity; and σ is the electrical conductivity (S/m).

**Figure 7:**
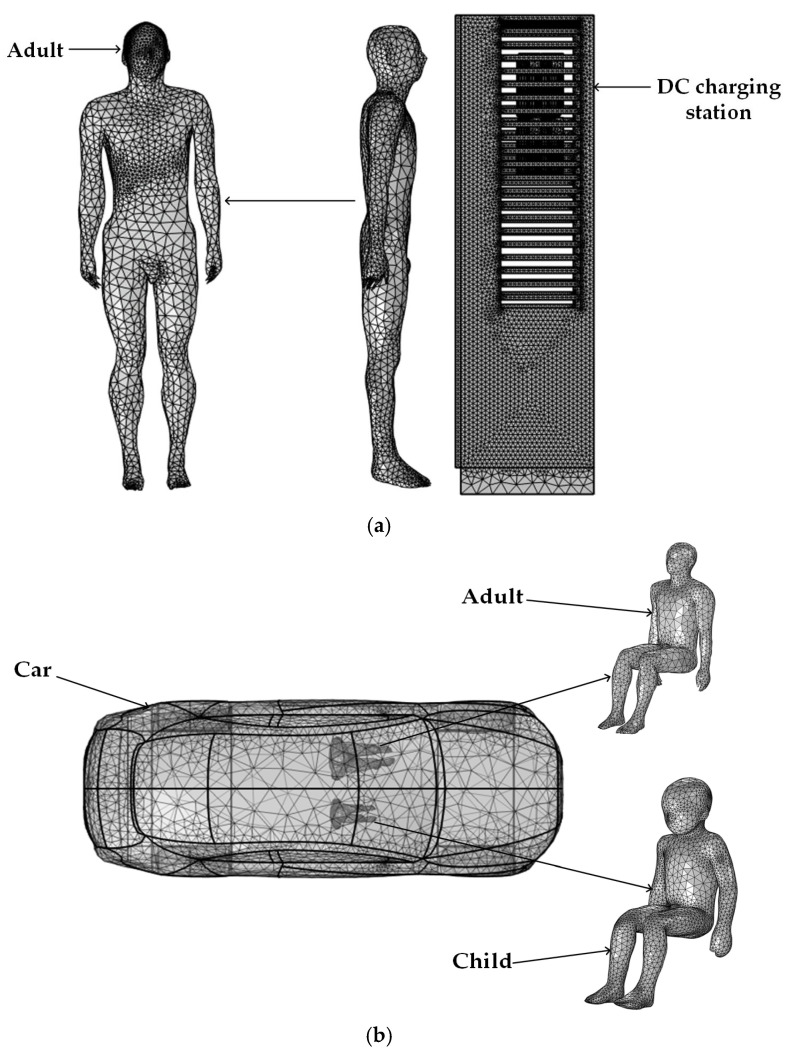
Mesh diagram of geometric models: (**a**) Adult and DC charging station; (**b**) Car, child and adult.

All numerical simulations were conducted on a desktop computer equipped with an Intel^®^ Core™ i7 processor and 64 GB of RAM. The three-dimensional geometric models, including the human body, DC charging station, internal charging modules, transformer, and electric vehicle, were discretized into tetrahedral elements using the COMSOL Multiphysics 6.2 finite element platform [47,48]. Based on the governing equations (Equations (2)–(8), the Magnetic Fields interface within the AC/DC Module of COMSOL Multiphysics was employed to solve for the spatial distributions of the electric field and magnetic field around the charging infrastructure and within the biological tissues of the passengers and driver. The human body, vehicle structure, and power electronics modules were meshed using fine-resolution tetrahedral elements, thereby improving the accuracy of field computation, particularly within anatomically sensitive regions. The mesh configuration of the computational domain is shown in Figure 7.

As DC fast charging technology for electric vehicles becomes increasingly widespread, both maintenance personnel and passengers may be subject to long-term exposure to electromagnetic fields in typical operational settings. To assess the potential biological effects, this study quantitatively evaluated the specific electromagnetic exposure levels in various organs and tissues of both technical staff and private passengers, thereby contributing to the occupational and public health risk assessment associated with high-power EV charging infrastructure.

### 2.5. ICNIRP Exposure Limits

To scientifically evaluate human electromagnetic exposure limits and protect public health, the International Commission on Non-Ionizing Radiation Protection (ICNIRP) and the Institute of Electrical and Electronics Engineers (IEEE) have established guidelines for human electromagnetic exposure limits [49,50,51,52]. This study adopts the occupational and public exposure limits outlined in the ICNIRP guidelines. The ICNIRP has defined exposure limits for occupational and public exposure across different frequency bands to protect both groups from health risks associated with electromagnetic exposure. For the frequency bands of 80 kHz, 85 kHz, 90 kHz, and 95 kHz, the exposure limits for the 3 kHz–100 kHz range are applied. Table 3 presents the occupational and public exposure limits for the 3 kHz–100 kHz frequency range.

This study evaluates the electromagnetic exposure levels for adults and children in various scenarios. Only the scenario involving an adult standing 0.1 m from the charging station considers both the ICNIRP occupational and public exposure limits, while all other scenarios consider only the ICNIRP public exposure limits.

## 3. Electromagnetic Exposure Safety Assessment

This study conducted numerical simulations of the **B**-field and **E**-field distributions within the bodies of occupational workers and private vehicle occupants of different age groups (including adults and children). At the 80 kHz frequency band, the electromagnetic exposure levels were systematically evaluated for adults and children at distances of 0.1 m, 0.3 m, and 0.6 m from the charging station, as well as for an adult in the driver’s seat and a child in the front passenger seat. Furthermore, by assessing the exposure levels in a typical scenario—an adult standing 0.1 m directly in front of the charging station—across multiple frequency bands, the electromagnetic field distributions in major human organs were analyzed. The results confirm the broad applicability of the study’s conclusions under different frequency conditions.

### 3.1. B-Field Distribution of the DC Charging Station

**Figure 8:**
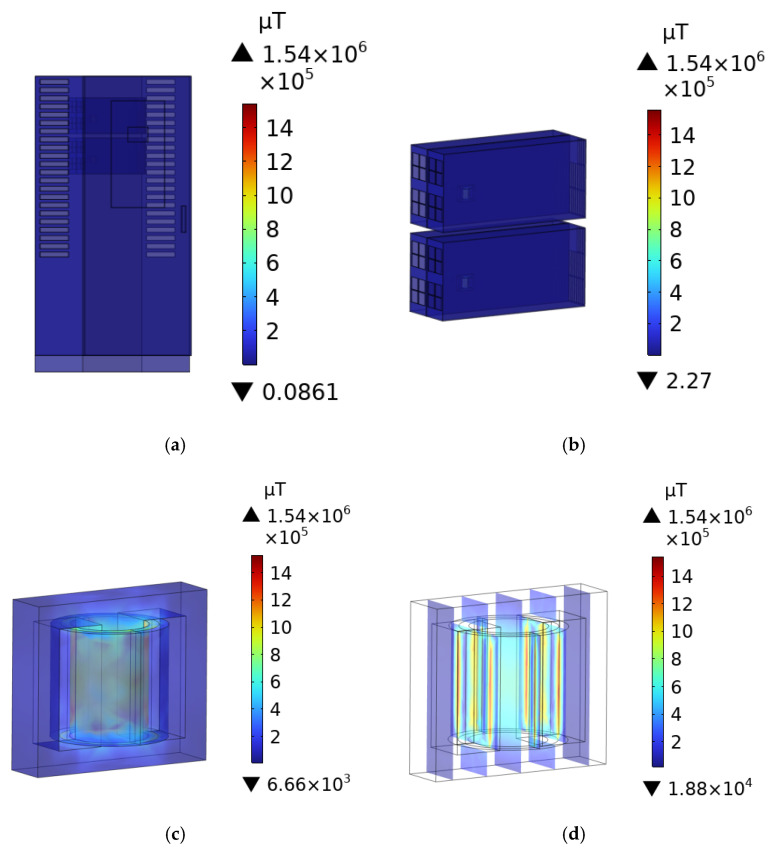
**B**-field distribution of the DC charging station and its components: (**a**) DC charging station body; (**b**) Charging module; (**c**) Transformer; (**d**) Transformer cross-section.

After the AC signal is input into the primary coil of the transformer, the secondary coil generates an induced electromotive force due to mutual inductance, which leads to the output of voltage signals that radiate an electromagnetic field. This paper presents only the distribution of the **B**-field within the transformer at a switching frequency of 80 kHz. Figure 8d illustrates the distribution of the induced **B**-field within the DC charging station, the charging modules, and the transformer. The maximum **B**-field was observed at the primary winding of the transformer, with a peak value of 1.54 × 10^6^ µT. As the distance from the transformer increases, the **B**-field diminishes.

### 3.2. **B**-Field Distribution Within Adult and Child at Different Distances

**Figure 9:**
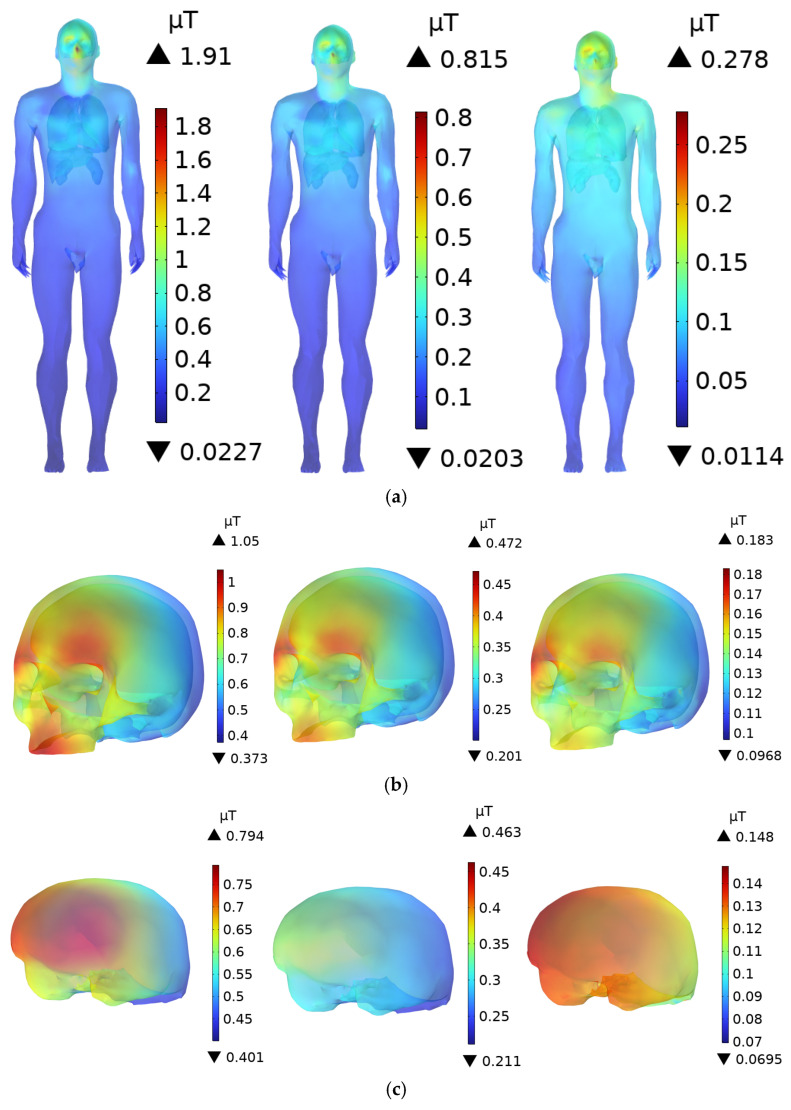

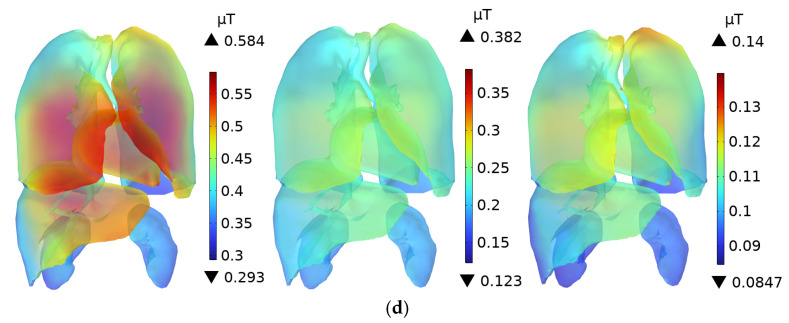
**B**-field distribution in an adult at distances of 0.1 m, 0.3 m, and 0.6 m from the DC charging station: (**a**) Whole Body; (**b**) Head; (**c**) Brain; (**d**) Internal organs.

In the magnetic field domain, the magnetic permeability of human tissues and organs is assumed to be equivalent to that of air [53,54]. Therefore, the **B**-field within the tissues is the same as that in the surrounding air, and the human body does not significantly alter the magnetic field distribution. Figure 9 shows the distribution of the **B**-field in the body and various organs of an adult at distances of 0.1 m, 0.3 m, and 0.6 m from the DC charging station. At a distance of 0.1 m from the DC charging station, the maximum **B**-field at the scalp is 1.91 µT, while the values for the skull and brain are 1.05 µT and 0.794 µT, respectively. The ICNIRP occupational exposure limit for the 3 kHz to 10 MHz frequency range is 100 µT, and the public exposure limit is 27 µT. The **B**-field at the scalp accounts for 1.91% and 7.07% of the ICNIRP occupational and public exposure limits, respectively. The magnetic flux densities in the other organs are well below the ICNIRP exposure limits.

**Figure 10:**
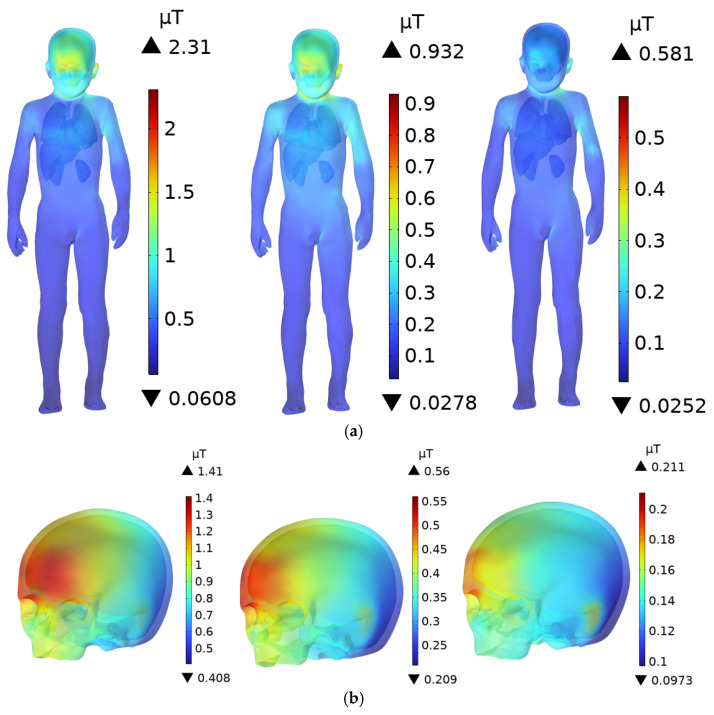

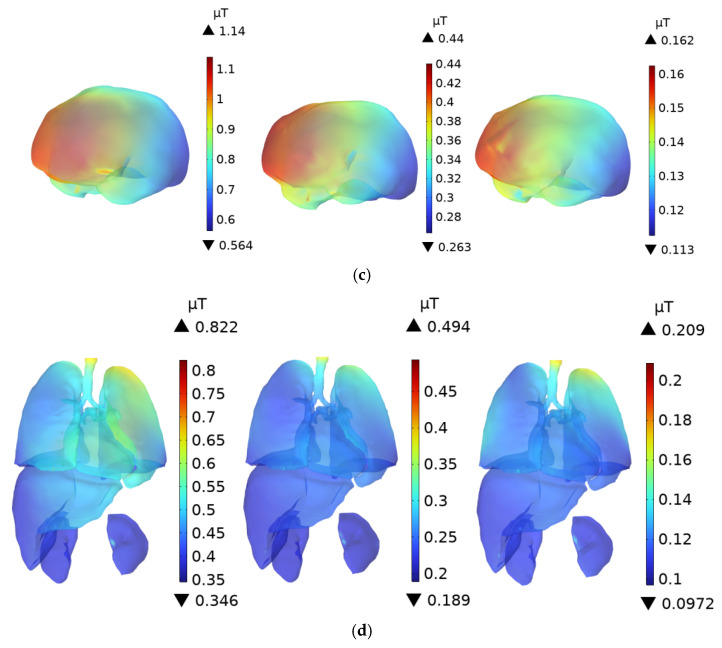
**B**-field distribution in a child at distances of 0.1 m, 0.3 m, and 0.6 m from the DC charging station: (**a**) Whole Body; (**b**) Head; (**c**) Brain; (**d**) Internal organs.

For children, due to their standing posture, which is slightly inclined forward, the distance to the radiation source is closer compared to adults, resulting in a slightly higher **B**-field. As shown in Figure 10, when a child stands 0.1 m from the DC charging station, the peak **B**-field at the scalp is 2.31 µT, while the peak values for the skull and brain are 1.41 µT and 1.14 µT, respectively. The peak **B**-field at the child’s scalp is 120.1% of that in the adult’s scalp, and it accounts for 8.56% of the ICNIRP public exposure limit, which is still significantly lower than the ICNIRP limits. The **B**-field in the other organs also remains well below the ICNIRP public exposure limits.

As the distance increases, the **B**-field decreases rapidly. At distances of 0.3 m and 0.6 m, the maximum **B**-field remains at the scalp. For adults, the maximum values of **B**-field are 0.815 µT and 0.278 µT, respectively, while for children, the corresponding values are 0.932 µT and 0.581 µT, all of which are below the ICNIRP exposure limits.

### 3.3. E-Field Distribution Within Adults and Children at Different Distances

**Figure 11:**
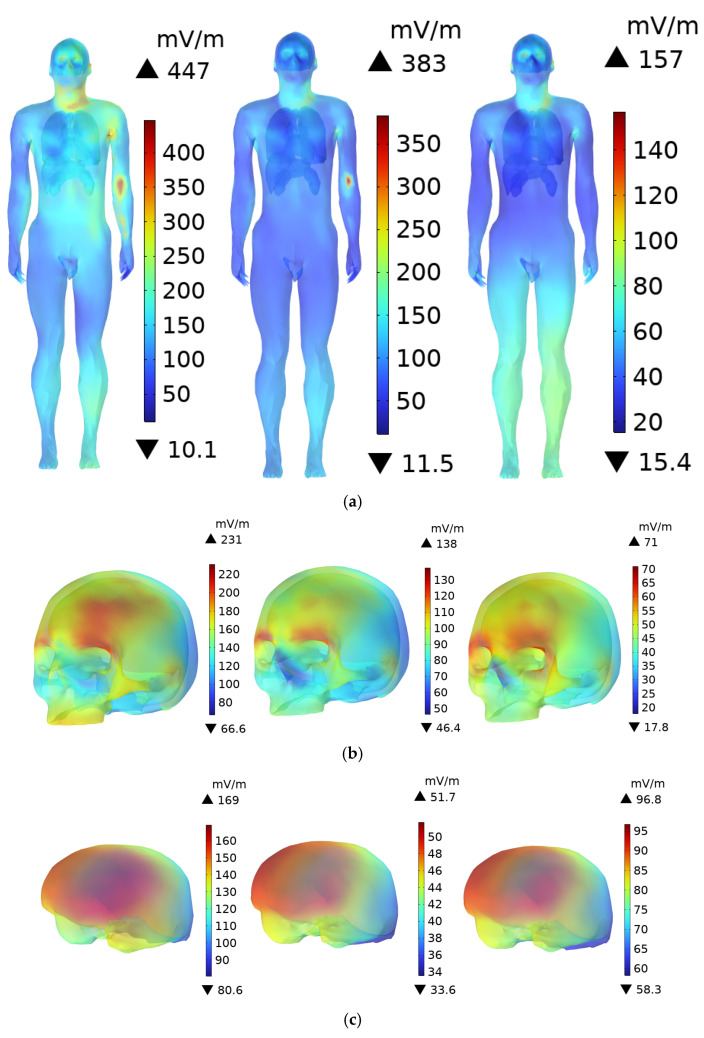

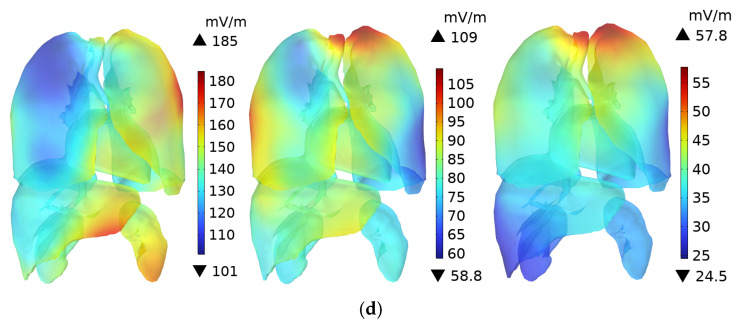
**E**-field distribution in an adult at distances of 0.1 m, 0.3 m, and 0.6 m from the DC charging station: (**a**) Whole Body; (**b**) Head; (**c**) Brain; (**d**) Internal Organs.

As shown in Figure 11, the DC charging station door is composed of a tempered glass screen and a steel plate. Since the conductivity of tempered glass is lower than that of steel, its shielding effect on the electric field is much weaker. The upper body of the adult human model faces the tempered glass section, while the four charging modules are located behind the tempered glass. Therefore, the head and upper body of the adult are directly exposed to the radiation from the source. When the individual is 0.1 m from the DC charging station, the peak **E**-field in the body occurs at the scalp, where it reaches 447 mV/m. The ICNIRP limits for **E**-field exposure in the 3 kHz to 10 MHz frequency range are 21,600 mV/m for occupational exposure and 10,800 mV/m for public exposure. The peak **E**-field at the scalp represents 2.01% and 4.13% of the ICNIRP occupational and public exposure limits, respectively. The peak **E**-field at the skull (231 mV/m) is higher than in the brain, with the skull facing directly toward the DC charging station. The peak **E**-field in the brain is 169 mV/m. As shown in Figure 11, the maximum **E**-field in the brain occurs in the anterior part, where the color is slightly redder compared to other regions. For the body, the lungs and liver are closer to the exposure source than the other organs, resulting in slightly higher **E**-field of 185 mV/m and 175 mV/m, respectively, at 0.1 m. The conductivity of the lungs is slightly higher than that of the liver, which explains the higher **E**-field in the lungs.

**Figure 12:**
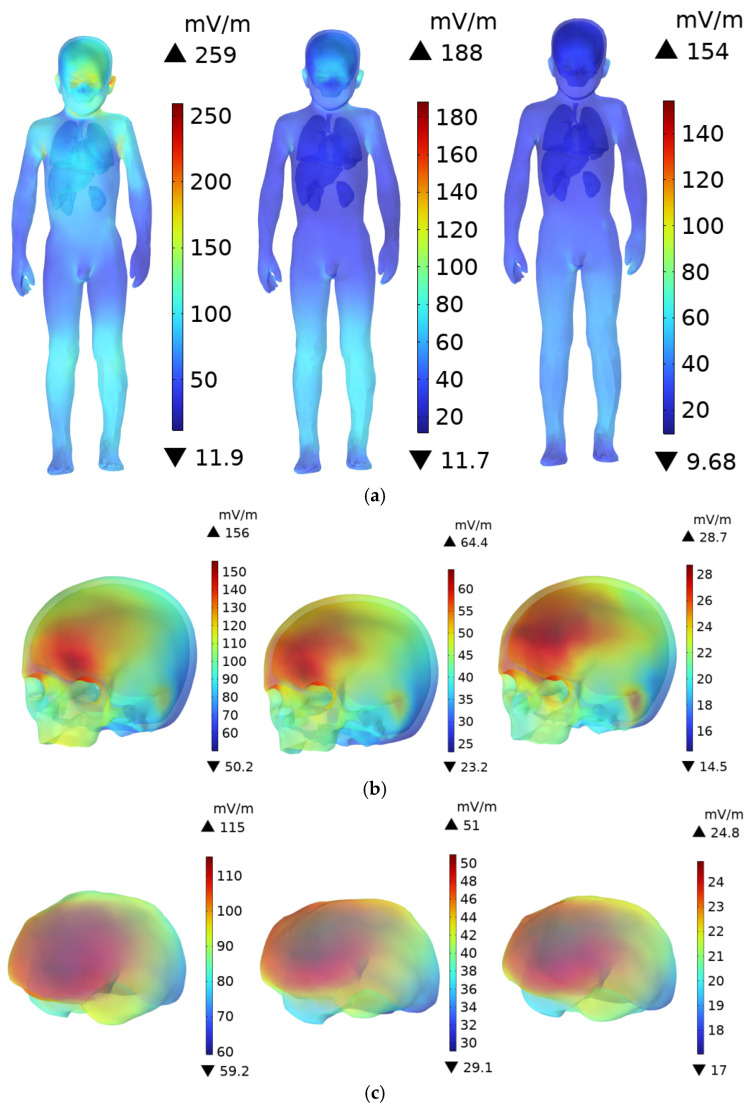

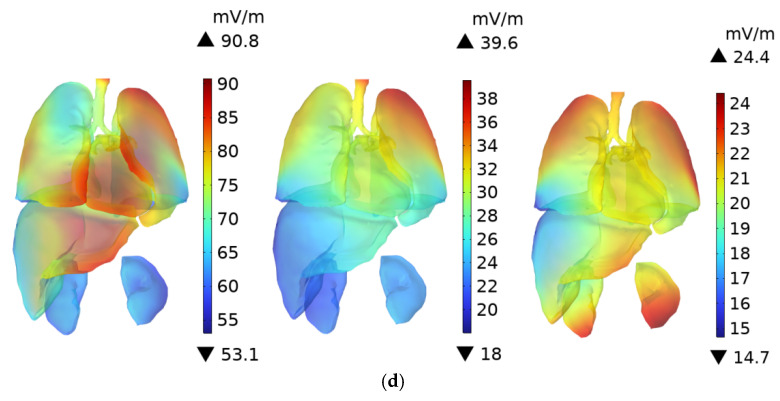
**E**-field distribution in a child at distances of 0.1 m, 0.3 m, and 0.6 m from the DC charging station: (**a**) Whole Body; (**b**) Head; (**c**) Brain; (**d**) Internal Organs.

For children, due to their shorter stature, the head and upper body face the steel plate door, which provides some shielding due to the higher conductivity of steel. Consequently, the **E**-field within the child’s body is lower than in the adult’s body. As shown in Figure 12, when the child stands 0.1 m from the DC charging station, the maximum **E**-field occurs at the scalp, with a value of 259 mV/m. This is 57.94% of the adult’s peak scalp **E**-field and 2.40% of the ICNIRP public exposure limit. The peak **E**-field in the child’s skull and brain are 156 mV/m and 115 mV/m, respectively.

As shown in Figure 11 and Table 4, as the distance increases, the **E**-field in the body and various organs decreases. At 0.1 m, the **E**-field are much higher than at 0.6 m, indicating that the electromagnetic exposure risk to the human body decreases with increasing distance from the DC charging station.

### 3.4. **B**-Field Distribution in Car

**Figure 13:**
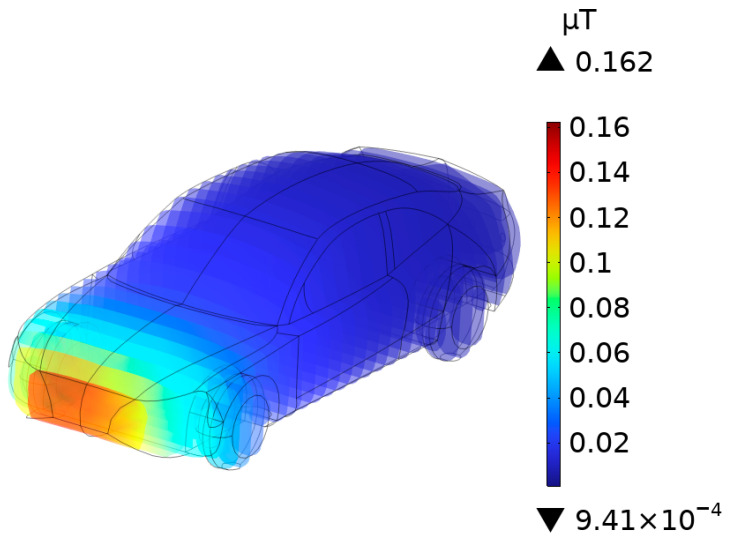
**B**-Field distribution in car.

The distribution of **B**-field induced by the internal transformer of a high-power DC charging station operating at 80 kHz was measured at an oblique lateral vertical distance of approximately 0.9 m. As shown in Figure 13, the maximum **B**-field occurs at the front part of the vehicle body closest to the DC charging station. In regions farther away from the DC charging station, the **B**-field gradually decreases with increasing distance.

### 3.5. Driver’s Body **B**-Field Distribution

**Figure 14:**
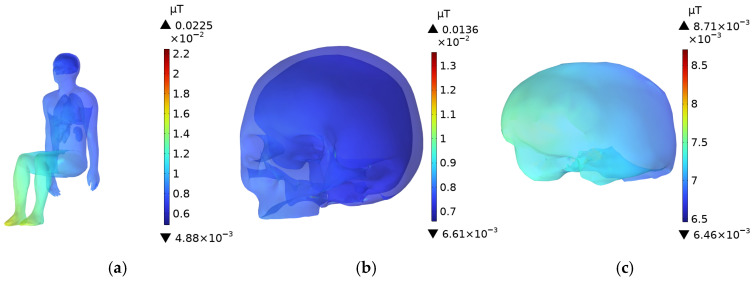
**B**-Field distribution of an adult in the driving sea: (**a**) Whole Body; (**b**) Head; (**c**) Brain.

**Figure 15:**
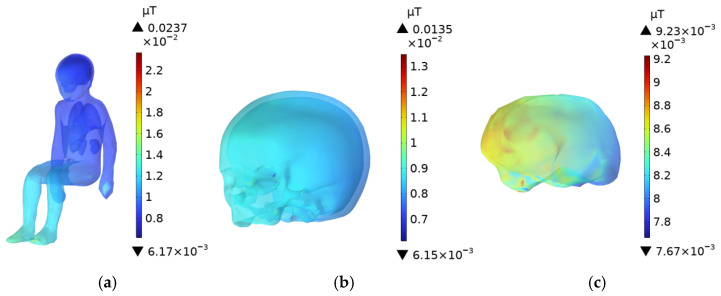
**B**-Field distribution of a child in the front passenger seat: (**a**) Whole Body; (**b**) Head; (**c**) Brain.

As shown in Figure 14, the **B**-field is highest in the legs and feet of the driver, as these body parts are closest to the DC charging station. Although the child in the passenger seat is slightly closer to the radiation source than the driver, the distance difference is not significant, and therefore, the **B**-field distribution in the child’s body is similar to that of the driver, as illustrated in Figure 15. The highest **B**-field in the child’s body remains in the feet and lower legs, which are closest to the DC charging station. Both the driver and the child’s head and brain are directly aligned with the radiation source, so the B-field in these areas are the highest.

### 3.6. Driver’s Body **E**-Field Distribution

**Figure 16:**
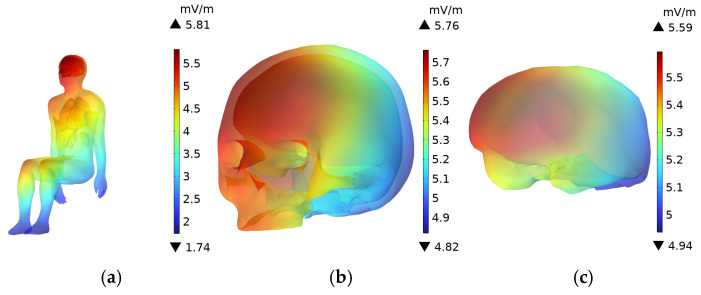
**E**-Field distribution of an adult in the driving seat: (**a**) Whole Body; (**b**) Head; (**c**) Brain.

**Figure 17:**
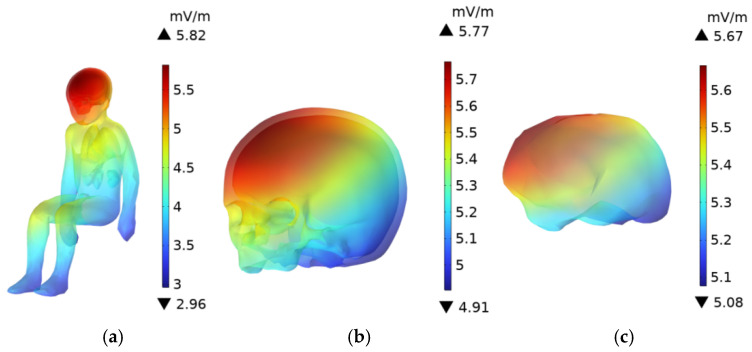
**E**-Field distribution of a child in the front passenger seat: (**a**) Whole Body; (**b**) Head; (**c**) Brain.

As shown in Figure 16 and Figure 17, the **E**-field is higher in the head, upper body, and knees, while the **E**-field at the feet is lower. The **E**-field in the upper body and knees is slightly lower than in the head. Since the distance between the driver and the passenger is relatively small, the distribution of the electric field in the driver’s and passenger’s bodies is quite similar. The skull and brain, which are directly exposed to the car’s glass, experience the least shielding from the electric field due to the low conductivity of the glass compared to the aluminum alloy car body. As a result, the **E**-field in these organs is higher than in other body parts. The peak **E**-field in the skull and brain of the adult are 5.78 mV/m and 5.61 mV/m, respectively, while the peak **E**-field in the child’s skull and brain are 5.77 mV/m and 5.67 mV/m.

Due to the greater distance between the driver and the DC charging station, along with the shielding effect of the aluminum alloy car body and windshield, the electric field distribution in the trunk organs is more uniform. Among the organs in the adult’s trunk, the lungs, being the closest to the radiation source, experience the highest electric field, with a peak value of 4.83 mV/m. The heart, with a relatively high dielectric constant, has a higher **E**-field than all organs except the lungs, with a peak value of 4.65 mV/m. The kidneys, being the furthest from the DC charging station in the seated position, have a slightly lower **E**-field compared to the liver, whose dielectric constant is similar, with a peak value of 3.99 mV/m.

**Figure 18:**
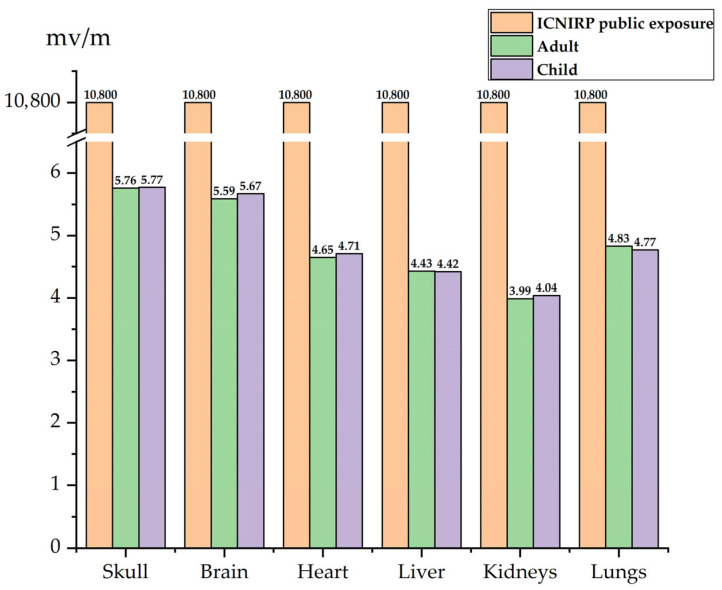
Comparison of the peak **E**-field in various organs of an adult and child with the ICNIRP public exposure limits.

The electric field peak values in the trunk organs of the driver and the child in the passenger seat are very similar, as shown in Figure 18, with a difference of no more than 2% between the maximum **E**-field in the organs of the two individuals.

### 3.7. Electromagnetic Exposure Levels at Different Frequencies

**Figure 19:**
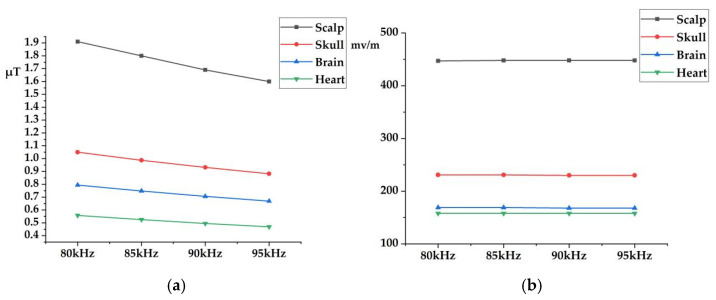
Electromagnetic Exposure Levels of Specific Organs at Different Frequencies: (**a**) Peak **B**-field; (**b**) Peak **E**-field.

The penetration depth and tissue absorption characteristics of electromagnetic waves vary with frequency. This section calculates the electromagnetic exposure levels within the body of an adult standing 0.1 m from the charging station across different frequency bands. Figure 19 illustrates the electromagnetic exposure levels in key organs, including the skin, brain, skull, and heart, at various frequencies. The peak **B**-field is consistently observed at the scalp, the organ closest to the radiation source, with values of 1.91 µT, 1.8 µT, 1.69 µT, 1.6 µT, and 1.52 µT, corresponding to 7.07%, 6.67%, 6.26%, 5.93%, and 5.63% of the ICNIRP occupational exposure limits, and 1.91%, 1.8%, 1.69%, 1.6%, and 1.52% of the ICNIRP public exposure limits, respectively. The peak **B**-field in various organs within the adult body decreases as the frequency increases.

As frequency increases, the electrical conductivity of biological tissues rises, while the dielectric constant decreases, leading to reduced penetration of the electric field. Due to the presence of multiple shielding layers in the charging station model (e.g., module casing and cabinet door) and the proximity of the evaluated frequencies, the variation in **E**-field across organs is relatively small. With increasing frequency, the variation in **E**-field within the human body is significantly smaller than that of **B**-field.

## 4. Conclusions

This study uses the transformer in the DC/DC circuit of a DC charging station module as the radiation source and employs realistic adult and child human models as exposure subjects. At an 80 kHz switching frequency, the electromagnetic field excited by the AC signal input to the primary winding was calculated, and electromagnetic exposure levels were assessed for individuals in proximity to the charging station and for an adult in the driver’s seat and a child in the front passenger seat during EV charging. The main conclusions are as follows:

(1) For an adult standing 0.1 m from the charging pile, the peak **B**-field (1.91 µT) and the peak **E**-field (447 mV/m) in the body are 1.91% and 2.07% of the ICNIRP occupational exposure limits, respectively, and 7.07% and 4.14% of the public exposure limits. For the child, the electromagnetic exposure levels (2.31 µT and 259 mV/m) are 8.56% and 2.40% of the public exposure limits, respectively. These results indicate that electromagnetic exposure levels for occupational and private individuals near the DC charging pile are within safe limits.
(2) For an adult standing at distances of 0.1 m, 0.3 m, and 0.6 m from the DC charging pile, the peak **B**-field in the body corresponds to 7.07%, 3.02%, and 0.80% of the ICNIRP public exposure limits, respectively, while the peak **E**-field corresponds to 2.07%, 1.77%, and 0.44%. For a child at the same distances, the peak **B**-field is 8.56%, 3.45%, and 2.15% of the ICNIRP public exposure limits, and the peak **E**-field is 2.40%, 1.74%, and 1.43%. These findings demonstrate a significant negative correlation between the distance from the charging pile and the electromagnetic exposure level in the body.
(3) During electric vehicle charging, the peak **B**-fields in the body for an adult in the driver’s seat and a child in the passenger’s seat are 0.0225 × 10^−2^ µT and 0.0237 × 10^−2^ µT, respectively. The peak **E**-fields in the body and brain are 5.81 mV/m and 5.59 mV/m for the adult, and 5.82 mV/m and 5.67 mV/m for the child, all significantly below the ICNIRP public exposure limits.
(4) In the typical scenarios (adult at 0.1 m from the charging pile), analyses were conducted at multiple frequency bands, including 85 kHz, 90 kHz, and 95 kHz. The results show that as the frequency increases, the peak **B**-field in the human body gradually decreases, while the **E**-field in the body shows no significant change, possibly influenced by the vehicle body. The electromagnetic exposure levels in all frequency bands are below the ICNIRP safety limits.

This study has certain limitations, and future research will focus on the following areas: (1) expanding the types of human models to include sensitive populations such as pregnant women and adolescent females; (2) evaluating electromagnetic exposure from multiple radiation sources (e.g., inverters and cables) acting concurrently; (3) conducting field measurements and validation of electromagnetic fields from DC charging stations.

This study addresses gaps in the safety assessment of electromagnetic environments associated with DC charging stations, providing a scientific basis for establishing electromagnetic environment standards and industry exposure limits for EV charging facilities, thereby contributing to public health protection.

## Figures and Tables

**Table 1:** Electrical conductivity and relative permittivity of the primary materials used in the model.

Main Materials of DC Charging Station and Electric Vehicle	Relative Permittivity	Conductivity (S/m)
Aluminum alloy (vehicle body)	1	2.33 × 10^7^
Tempered glass	5.5	1
Copper (transformer coil)	1	6 × 10^7^
Ferrite (transformer core)	10	1.54 × 10^−1^

**Table 2:** Dielectric properties and conductivity of various human tissues and organs.

Human Body Tissue	Relative Permittivity				Conductivity (S/m) (80 kHz)			
	80 kHz	85 kHz	90 kHz	95 kHz	80 kHz	85 kHz	90 kHz	95 kHz
Skin	17598	16995	16421	15876	0.05211	0.05568	0.05915	0.06254
Skull	291.607	285.35	279.74	274.65	0.03590	0.03592	0.03595	0.03597
Brain	3140.9	3002.4	2878.2	2766.2	0.10601	0.11034	0.10691	0.10735
Heart	11670	4325.8	10662	10233	0.20850	0.21027	0.21195	0.21356
Liver	8381.7	11137	7903.1	7693.1	0.07988	0.08108	0.08225	0.08342
Kidney	8637.6	8131.6	8095.4	7863.1	0.16714	0.16823	0.16930	0.17033
Lung	4516	8352.1	4155.6	4002.3	0.18705	0.18770	0.18831	0.18891

**Table 3:** Electromagnetic Exposure Limits at 80 kHz.

Electromagnetic Exposure Limits at 80 kHz			
Exposure Category	Frequency Range	Electric Field (mV/m)	Magnetic Induction Intensity (µT)
Occupational	3 kHz–10 MHz	21,600	100 µT
General public	3 kHz–10 MHz	10,800	27 µT

**Table 4:** Maximum **E**-Field Values of Various Tissues and Organs at Different Distances.

Organs	E(mv/m)					
	The Distance Between the Person and the DC Charging Station is 0.1 m		The Distance Between the Person and the DC Charging Station is 0.3 m		The Distance Between the Person and the DC Charging Station is 0.6 m	
	Adult	Children	Adult	Children	Adult	Children
Skull	231	156	138	64.4	71	28.7
Brain	169	115	96.8	51	51.7	24.8
Heart	158	90.4	102	31.6	41.7	21.6
Liver	175	87.7	92.1	28.7	38.4	22.3
Kidney	147	65.3	81.1	24	32.8	24.4
Lung	185	90.8	109	39.6	57.8	24.4

## Data Availability

The data in this study are available from the corresponding author upon request.
